# Novel insights into the lipid signalling in human spermatozoa

**DOI:** 10.1093/humrep/deaf085

**Published:** 2025-05-23

**Authors:** Steven Serafini, Cristian O’Flaherty

**Affiliations:** Experimental Medicine Division, Department of Medicine, McGill University, Montréal, QC, Canada; Urology Division, Department of Surgery, McGill University, Montréal, QC, Canada; The Research Institute, McGill University Health Centre, Montréal, QC, Canada; Experimental Medicine Division, Department of Medicine, McGill University, Montréal, QC, Canada; Urology Division, Department of Surgery, McGill University, Montréal, QC, Canada; The Research Institute, McGill University Health Centre, Montréal, QC, Canada; Department of Anatomy and Cell Biology, McGill University, Montréal, QC, Canada; Department of Pharmacology and Therapeutics, McGill University, Montréal, QC, Canada

**Keywords:** male factor infertility, sperm capacitation, acrosome reaction, lipidomics, redox signalling, lipid signalling

## Abstract

Infertility, affecting one in six couples worldwide, poses significant health and social challenges. While both male and female factors contribute to infertility, male infertility causes remain underexplored, with about 34% of cases classified as unexplained. A few studies focus on the role of lipids in male fertility, and some lipids are rising as key players in spermatozoa. This review highlights the importance of lipids, particularly phospholipids, neutral lipids, and glycolipids, in spermatozoa during capacitation and the acrosome reaction (AR). The dynamic lipid profile of human spermatozoa is crucial for their development, maturation, and fertilization capability. During epididymal maturation, sperm undergo crucial biochemical changes, including increased production of phosphatidylcholine and sphingomyelin, which enhance membrane integrity and mobility. Increased levels of ceramide affect membrane fluidity and signalling necessary for sperm function. As spermatozoa enter the female reproductive tract, they adjust their lipid content for capacitation and fertilization. Lipid signalling is crucial for human spermatozoa, influencing their viability and fertilization potential during transit through the female reproductive tract. Lysophosphatidic acid, abundant in seminal plasma, enhances sperm motility, facilitates the AR by promoting glycolysis and calcium influx, and is important for maintaining sperm viability. The remodelling of lipid rafts, enriched in cholesterol and sphingolipids, is essential for signal transduction and capacitation. Sphingolipids, particularly sphingosine 1-phosphate and ceramide, play significant roles in sperm capacitation and AR by promoting reactive oxygen species production and calcium signalling, respectively. Understanding these lipid dynamics will increase our knowledge of the complexity of sperm metabolism.

## Graphical abstract

**Graphical Abstract:**
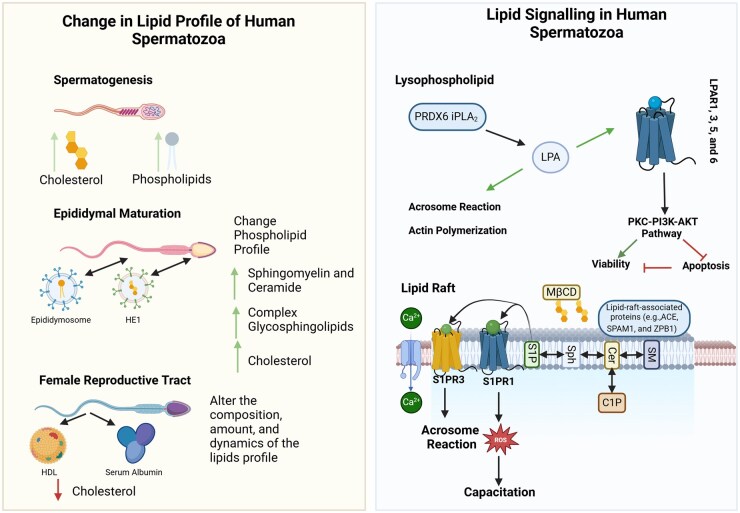
**Lipid profile and signalling in human spermatozoa**. Created in BioRender. Lab, O. (2025) https://BioRender.com/6ql7m63

## Introduction

Infertility is a noteworthy health and social concern affecting one in six couples globally (Collins, 2019; World Health Organization, 2021). Over the past few decades, global infertility rates have increased due to several factors, including delayed parenthood as couples postpone conception for career advancement and financial stability (Schmidt *et al.*, 2012). As individuals age, fertility declines for both men (Zitzmann, 2013) and women (Baird *et al.*, 2005), reducing the chances of conception. Additionally, lifestyle factors such as poor diet, smoking, alcohol consumption, high stress, lack of exercise, and insufficient sleep can negatively affect fertility (Sharma *et al.*, 2013). Exposure to environmental chemicals, including pesticides, plastics, and pollutants, can interfere with hormonal balance and reproductive function, potentially reducing fertility rates (Ghosh *et al.*, 2022).

Although both male and female factors contribute to rising infertility rates (Jung and Seo, 2014; Kumar and Singh, 2015), women have historically borne the stigma and treatment burden (Turner *et al.*, 2020). Meanwhile, several studies have noted a decline in global semen quality in recent decades (Aitken, 2013; Kumar and Singh, 2015). In addition, ∼34% of male infertility cases have normal semen analysis yet remain unexplained due to our limited understanding of the molecular mechanisms behind male fertility and the reliance on conventional semen analysis, which only assesses basic parameters like concentration, motility, morphology, hormone levels, and, in a lesser proportion, DNA fragmentation, lacks accuracy in pinpointing the cause of male infertility (Turner *et al.*, 2020). So far, few efforts have been made to develop new tools for identifying sperm dysfunction.

Triglycerides and phospholipids are key components of spermatozoa (Schisterman *et al.*, 2014; Ferramosca *et al.*, 2018). Normozoospermic infertile men show altered lipid profiles and increased lipid peroxidation (Oborna *et al.*, 2010) and reduced motility and sperm count, particularly with lower levels of very-long-chain polyunsaturated fatty acids (VLC-PUFAs) (Craig *et al.*, 2019). Approximately 24% of the human sperm proteome is linked to lipid metabolism (Amaral *et al.*, 2014). This evidence highlights the need for renewed research into lipidomics and its role in sperm function, including protection against oxidative stress, maintaining viability, and supporting processes like capacitation and acrosome reaction (AR). Understanding sperm dysfunction and its role in idiopathic infertility could pave the way for new diagnostic tools and therapies for male infertility.

This review explores the functional roles of lipids in human and mammalian spermatozoa during fertilization, emphasizing their critical importance in maintaining male fertility.

## The major lipid class in sperm membranes

Studies have characterized the lipid profiles of spermatozoa across various mammalian species, including mice (Alvarez *et al.*, 1987), boars, and humans (Leßig *et al.*, 2004). Although there are species-specific differences, spermatozoa typically contain about 70% phospholipids, 25% neutral lipids, and 5% glycolipids (Mann and Lutwak-Mann, 1981). Recently, a detailed breakdown of human sperm lipid composition was reviewed (Shan *et al.*, 2021). Phospholipids are central to sperm function, with ∼24% of sperm proteins involved in lipid metabolism (Amaral *et al.*, 2014). Our research further investigates key lipids critical for sperm function and fertility.

### Phospholipids

**Figure 1.**
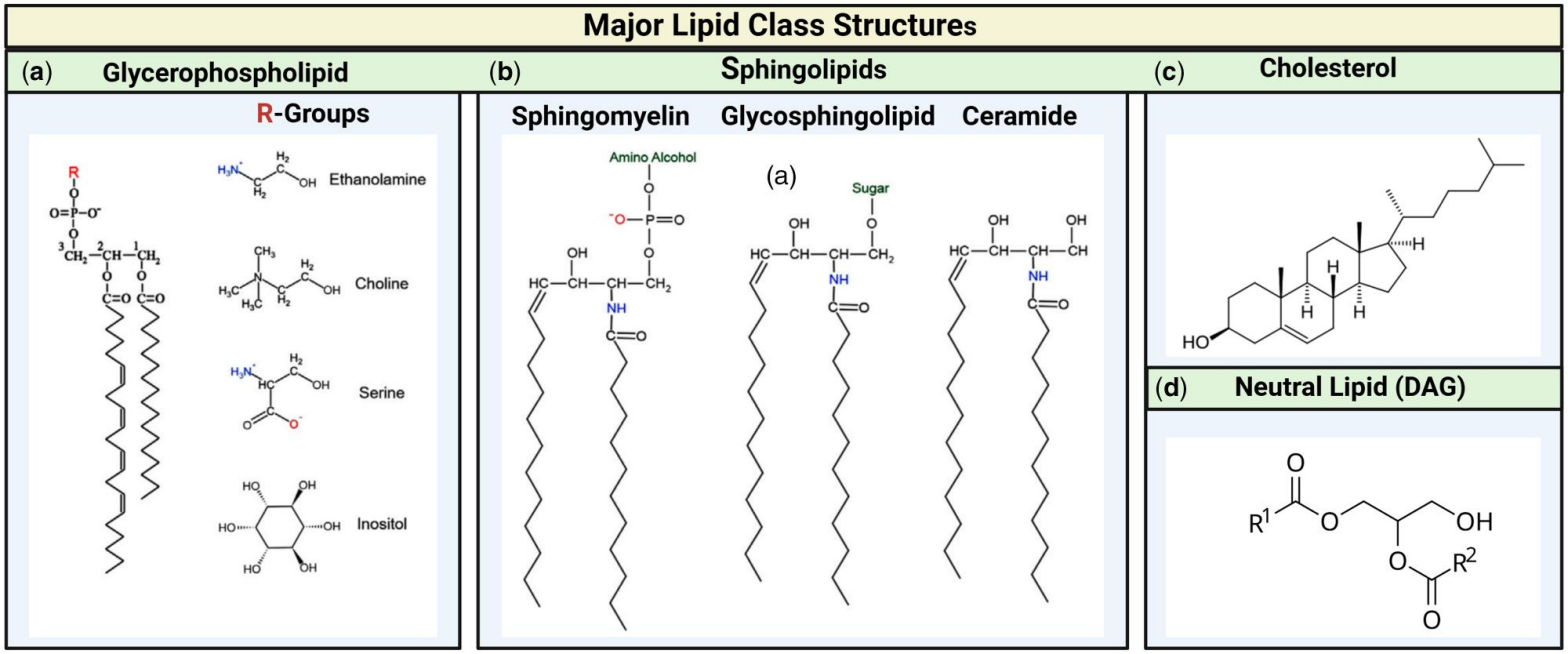
**Structure of the major lipid classes in spermatozoa.** This diagram illustrates the key lipid components found in sperm cells, highlighting their molecular structures. The major lipid classes depicted include (**a**) Glycerophospholipids, (**b**) sphingolipids, (**c**) cholesterol, and (**d**) diacylglycerol (neutral lipid), each contributing to the structural integrity and functionality of the sperm membrane. This figure was created in BioRender. Lab, O. (2025). https://BioRender.com/c82x050.

Phospholipids with a glycerol backbone are classified as glycerophospholipids (GPLs), the primary structural lipids in eukaryotic membranes. They consist of two fatty acids (FA) esterified at the sn-1 and sn-2 positions of glycerol and a hydrophilic phosphate group at the sn-3 position (Fig. 1a) (Castro-Gómez *et al.*, 2015). The most common GPLs are phosphatidylcholine (PC) (Rodríguez‐Martínez *et al.*, 2011), phosphatidylethanolamine (PE), phosphatidylinositol, and phosphatidylserine (Gulaya *et al.*, 2001; Van Meer *et al.*, 2008).

Sphingolipids are distinct structural lipids vital for maintaining cell membrane integrity. Ceramide is the hydrophobic backbone of major sphingolipids in mammalian cells, including sphingomyelin and glycosphingolipids (Fig. 1b). Glycosphingolipids contain mono-, di-, or oligosaccharides derived from glucosylceramide and occasionally galactosylceramide (Fig. 1b) (Van Meer *et al.*, 2008).

Upon induction by reactive oxygen species (ROS) and enzymes, glycerolipids and sphingolipids undergo hydrolysis, producing various messenger lipids. These include lysophosphatidylcholine (LPC), lysophosphatidic acid (LPA), phosphatidic acid (PA), and diacylglycerol (DAG), as well as sphingosylphosphorylcholine, sphingosine, S1P, ceramide-1-phosphate (C1P), and ceramide (Van Meer *et al.*, 2008). LPC, LPA, SPC, sphingosine, and S1P contain a single aliphatic chain and can easily detach from membranes and signal through associated membrane receptors (Zu Heringdorf and Jakobs, 2007). On the contrary, PA, DAG, C1P, and ceramide remain in the membrane and can recruit cytosolic proteins for signal transduction (Fernandis and Wenk, 2007).

### Cholesterol and neutral lipids

Cholesterol is an amphipathic molecule that stabilizes the lipid bilayer of biomembranes. Neutral lipids include di- and tri-glycerol and cholesteryl esters. Of these neutral lipid classes, only DAG is present in sperm cells under certain circumstances, as sperm cells do not carry lipid droplets. Cholesterol is considered amphipathic due to its hydrophobic steroid backbone and a polar hydroxyl group, allowing it to interact with hydrophobic and hydrophilic regions of the membrane (Fig. 1c). In contrast, DAG is primarily a neutral lipid, consisting of a glycerol backbone with two FA chains, hence not amphipathic (Fig. 1d). Despite this, DAG plays a crucial role in membrane dynamics and signal transduction in the sperm membrane. The composition of neutral lipids varies between species and among individual males, and across different ejaculates within the same species (Mann and Lutwak-Mann, 1981; Cross, 1998). In human sperm, cholesterol represents up to 40% of total lipids, compared to just 22% in boar sperm (Parks and Lynch, 1992). Research has focused on cholesterol concentrations in the seminal plasma of animals such as rams, boars, stallions, humans, and domestic cats (Eliasson, 1966; Parks and Lynch, 1992; Garcia *et al.,* 2020), highlighting cholesterol’s important role in reproductive physiology and its potential impact on fertility (De Neergaard *et al*., 2018).

Cholesterol efflux from the sperm membrane is crucial for capacitation, a process embodying physiological and biochemical changes that the sperm undergoes after ejaculation, preparing them for fertilization. Removal of cholesterol from the sperm membrane alters membrane fluidity, destabilizes specific proteins, and primes the sperm for the AR (Travis and Kopf, 2002). Various ATP-binding cassette (ABC) proteins (e.g. ABCA1–3) that transport cholesterol to high-density lipoproteins (HDL) for removal or recycling are well established (Yvan-Charvet *et al.*, 2010). In boar spermatozoa, cholesterol efflux is facilitated by ABC Transporters, ABCA14 and ABCA17, as these proteins are among the top 5% in abundance based on total peptide count (Byrne *et al.*, 2012). After cholesterol is transported across the cell membrane, it is carried by proteins like albumin or HDL found in the female genital tract (Leahy and Gadella, 2015). While the oviduct contains high concentrations of albumin (Ehrenwald *et al.*, 1990), it mainly binds to FA, reducing its ability to accept cholesterol (Leahy and Gadella, 2015). Therefore, HDL is the primary cholesterol acceptor in the oviduct.

The synthesis and metabolism of DAG in cells involve complex biochemical processes (Cui and Houweling, 2002). During capacitation, the spermatozoon undergoes biochemical and plasma membrane lipid composition modifications essential for the AR. A key lipid-signalling molecule in this process is DAG, produced by phosphoinositide-specific phospholipase C (PLC) cleaving phosphatidylinositol 4,5-bisphosphate. This signalling cascade regulates the mobilization of intracellular calcium and the activation of protein kinase C (PKC); both are essential for initiating the AR (Lopez *et al.*, 2012).

### Glycolipids

Glycolipids are an important class of lipids found in sperm membranes, comprising ∼5–8% of total polar lipids in mammals (Parks and Lynch, 1992). Glycosphingolipids are made by attaching glycosidic head groups to ceramides. A notable example is seminolipid, a specific glycolipid with sulfogalactosylglycerol in its structure. Sulfogalactosylglycerolipid, or seminolipid, is the predominant anionic glycolipid found exclusively in the outer lipid leaflet of the sperm plasma membrane (and in Schwann cells). It is a highly abundant lipid in sperm, accounting for ∼8% of the total lipid content (Vos *et al.*, 1994). The molecular diversity of seminolipid is low, with the sn-1 alkyl group being predominantly palmityl and the sn-2 acyl group being palmitoyl. This contrasts with the high degree of unsaturation observed in PC and PE (Vos *et al.*, 1994). Interestingly, seminolipid is not homogeneously distributed across the sperm surface; rather, it undergoes a reorganization during sperm capacitation, shifting from the apical to the equatorial region of the sperm head, which is in a retrograde movement relative to cholesterol (Vos *et al.*, 1994).

## Dynamic lipid profile during human sperm development and their functional relevance

### Spermatogenesis

Phospholipids are essential for sperm development, maturation, and fertilization. An increase in lipid droplets in different cell types occurs during spermatogenesis (Osuga *et al.*, 2000). Lipid droplets have a role in Leydig cells, providing cholesterol for steroidogenesis (Freeman and Ascoli, 1982), and in Sertoli cells, they transfer cholesterol and phospholipids to spermatocytes (Kerr and De Kretser, 1975). These phenomena highlight the link between lipid metabolism and fertility during spermatogenesis. FAs accumulate in testicular cells via passive diffusion across the lipid bilayer and protein-facilitated transport mediated by the CD36 glycoprotein, which is highly expressed in Sertoli cells (Rato *et al.*, 2014). During spermatogenesis, the elongating spermatid’s volume decreases to 25% of its original size due to the loss of cytoplasm and various organelles, making them transcriptionally inert and metabolically dormant. However, further maturation in the epididymis is necessary for the sperm to acquire fertilization capability.

### Epididymal maturation

The epididymis is vital for maturing and storing spermatozoa. During the epididymal transit, sperm undergo key biochemical and biophysical changes that enable motility and fertilizing ability (Sommer *et al.*, 1996; Robaire and Hamzeh, 2011). In addition to changes in protein composition and increased negative surface charge, a key aspect of sperm maturation is the alteration of the plasma membrane’s lipid composition (Sullivan, 1999; Cuasnicú *et al.*, 2002).

The epididymal epithelium produces exosomes called epididymosomes (Yanagimachi *et al.*, 1985; Sullivan *et al.*, 2005; Rejraji *et al.*, 2006). Epididymosomes are rich in sphingolipids and PUFAs, particularly sphingomyelin and arachidonic acid (Table 1) (Frenette *et al.*, 2006; Rejraji *et al.*, 2006). They also carry GPI-anchored proteins, such as CD52, in humans (Yeung *et al.*, 2001). HE1, a small and soluble glycoprotein that binds cholesterol, is also found in the epididymis (Okamura *et al.*, 1999; Légaré *et al.*, 2006). Both epididymosomes and HE1 have roles in exchanging lipid contents and forming sperm membrane structures, such as lipid rafts (Frenette *et al.*, 2006).

**Table 1.** Changes in the profile of major lipid classes and their role in fertilization.

Lipid class	Major findings	Species	References
Sphingolipids	Epididymosomes rich in sphingomyelin, enhance membrane integrity and lipid raft formation.	Mouse Bovine	Rejraji *et al.* (2006) Frenette* et al.* (2006)
Phosphatidylcholine	It increases during maturation and is essential for membrane integrity and motility. Lysophosphatidylcholine enhances AR.	Mammals Mouse Human Dog Hamster	Sullivan and Saez (2013) Björkgren *et al.* (2015) Haidl and Opper (1997) Lucio *et al.* (2017) Llanos *et al.* (1993)
Phosphatidylethanolamine	Decreases during maturation and aids in fusion events.	Mouse Human	Rejraji *et al.* (2006) Gulaya *et al.* (2001)
Phosphatidylserine	It decreases during maturation and translocates to the outer leaflet, facilitating oocyte recognition and enhancing AR when exposed (via A23187).	Human Boar Boar Human	Haidl and Opper (1997) Flesch and Gadella (2000) Gadella and Harrison (2002) Martin *et al.* (2005)
Sphingomyelin	Increased level and translocate to outer leaflet, enhancing AR and oocyte recognition.	Rat Boar Mouse Human	Furland *et al.* (2007) Nikolopoulou *et al.* (1985) Rejraji *et al.* (2006) Nixon *et al.* (2011)
Ceramide	Produced from sphingomyelin hydrolysis, ceramide. Involved in signalling pathways for AR.	Rat Rat Mouse Boar Human	Zanetti *et al.* (2010) Furland *et al.* (2007) Björkgren *et al.* (2015) Murase *et al.* (2004) Vaquer *et al.* (2020)
Glycosphingolipids	Gangliosides increase during epididymal transit. Gangliosides (GM1, GD1a) facilitate sperm egg recognition, contributing to sperm functionality and fertility.	Mammals Rat Murine	Liu (2016) Oresti *et al.* (2011) Kawano *et al.* (2008)

AR, acrosome reaction; GM1, ganglioside M1; GD1a, ganglioside D1a.

Phospholipid composition in maturing spermatozoa changes during epididymal transit. There are unusually high levels of sn-2 22:6 (docosahexaenoic acid) and 22:5 (docosapentaenoic acid) esterified to PC and PE, with mostly sn-1 16:0 (palmitic acid) FAs and 16:0 ether plasminogen in testicular spermatozoa. The removal of the cytoplasmic droplet during sperm maturation appears to alter the overall lipid composition rather than lipid modifications (e.g. elongation or desaturation) in the epididymis. The epididymal maturation enhances membrane fluidity and supports sperm motility (Rejraji *et al.*, 2006; Gervasi and Visconti, 2017). The unsaturated FAs are also incorporated into phospholipids (Ramos Angrimani *et al.*, 2017). PC, a major phospholipid, increases in abundance during epididymal maturation and has a notable role in membrane integrity (Sullivan and Saez, 2013; Björkgren *et al.*, 2015), motility (Haidl and Opper, 1997; Lucio *et al.*, 2017), and its conversion product LPC enhances AR (Llanos *et al.*,1993) (Table 1).

PE levels decrease during transit, contributing to anisotropy and facilitating fusion events (Gulaya *et al.*, 2001; Rejraji *et al.*, 2006) (Table 1). Additionally, phosphatidylserine content decreases during maturation (Haidl and Opper, 1997). Notably, evidence has shown that the remaining phosphatidylserine translocates from the inner to the outer leaflet of the plasma membrane to increase recognition by the oocyte (Flesch and Gadella, 2000; Gadella and Harrison, 2002). In addition, phosphatidylserine exposure in human sperm, as induced by A23187, facilitates the AR (Martin *et al.*, 2005). Phospholipid changes during epididymal transit are crucial for sperm maturation, enabling improved binding to the zona pellucida (ZP) and enhanced fertilization rates.

Sphingomyelin and ceramide show significant alterations during epididymis maturation (Furland *et al.*, 2007). Increased sphingomyelin levels (Nikolopoulou *et al.*, 1985; Rejraji *et al.*, 2006) enhance membrane integrity and lipid raft formation, enabling specific proteins and receptors in these microdomains to facilitate signal transduction during capacitation and the AR (Van Gestel *et al.*, 2005; Tsai *et al.*, 2007; Nixon *et al.*, 2011; Watanabe and Kondoh, 2011; Lucchesi *et al.*, 2016; Ramal Sanchez *et al.*, 2018).

Ceramide, a key metabolite, is produced through hydrolysing sphingomyelin by sphingomyelinase during epididymal maturation. It greatly impacts membrane properties, such as detergent resistance and mechanical properties, including rigidity (Alonso and Goñi, 2018). Additionally, ceramide is involved in signalling pathways essential for acquiring fertilizing potential (Murase *et al.*, 2004; Zanetti *et al.*, 2010; Vaquer *et al.*, 2020) (Table 1). Glycosphingolipids, with a ceramide backbone and sugar residues, include neutral glycosphingolipids (e.g. cerebrosides) and acidic glycosphingolipids (e.g. gangliosides).

Complex glycosphingolipids, like gangliosides, increase during epididymal transit (Oresti *et al.*, 2011). These glycosphingolipids contribute to structural integrity and the forming of lipid rafts (Kawano *et al.*, 2008; Liu, 2016), and gangliosides such as GM1 and GD1a facilitate sperm-egg recognition (Kawano *et al.*, 2008) (Table 1). Changes in sphingomyelin, ceramide, and glycosphingolipids during epididymal maturation are essential for sperm functionality and fertility (Dacheux and Dacheux, 2014).

### Female reproductive tract

Upon entering the female reproductive tract, spermatozoa first encounter vaginal fluid, then interact with cervical mucus, which selects sperm based on kinetic efficiency and morphology (Jeulin *et al.*, 1985). Cervical mucus selects spermatozoa based on lipid content or alters the composition, amount, and dynamics of the lipids in the sperm plasma membrane, which are crucial for fertilization (Perry *et al.*, 1996; Feki *et al.*, 2004). In the oviduct, spermatozoa undergo capacitation and the AR before fertilization (Yanagimachi, 1994). Sperm capacitation, occurring at the oviduct’s isthmus, is a complex process involving biochemical changes, including the tightly regulated production of ROS, such as superoxide anion (O_2_^•−^), hydrogen peroxide, and nitric oxide (NO) (De Lamirande and O’Flaherty, 2012; O’Flaherty, 2015), timely phosphorylation events (De Lamirande and O’Flaherty, 2012; De Jonge, 2017; Serafini and O’Flaherty, 2022), and changes in the plasma membrane lipid contents (Cross, 1998). Capacitation is needed for subsequent hyperactive motility, ZP recognition, AR, and oocyte fertilization (Yanagimachi, 1994; De Jonge, 2017; Serafini and O’Flaherty, 2022). The process of sperm capacitation has been known since the 1950s (Chang, 1951; Austin, 1952), and some proteins (e.g. ion channels, protein kinases, etc) involved in its molecular mechanism have been described (De Jonge, 2017). Capacitation causes lipid profile changes in the plasma membrane, including loss of asymmetric distribution and reduced cholesterol and phospholipids (Davis, 1980, 1981; Fleming and Yanagimachi, 1981). Elevated levels of albumin and HDL in uterine and follicular fluid coincide with cholesterol redistribution to the sperm head’s apical regions and subsequent efflux (Leahy and Gadella, 2015). Cholesterol efflux increases the membrane’s permeability to calcium, bicarbonate, and potassium (Cross, 1998). Phospholipid levels decrease during capacitation as their hydrophobic tails break, forming lysophospholipids and FAs. These changes enhance motility and viability and promote AR (Zanetti *et al.*, 2010). During capacitation and the AR, there is an increase in hydrolysation of sphingomyelin into ceramide species with VLC-PUFAs (Zanetti *et al.*, 2010).

As mentioned above, the glycolipid seminolipid is not homogeneously distributed across the sperm surface; rather, it undergoes a reorganization during sperm capacitation, shifting from the apical to the equatorial region of the sperm head, which is in a retrograde movement relative to cholesterol (Gadella *et al.*, 1994; Gadella and Jovin, 1995; Flesch *et al.*, 2001). Seminolipid plays a dual and paradoxical role in the sperm cell. Before capacitation, the seminolipid oriented at the apical ridge may prevent preliminary fusion with the outer acrosomal membrane (OAM). After capacitation, the seminolipid reorients to the equatorial region, stabilizing this part of the plasma membrane and facilitating the final fusion between the sperm and egg cell membranes (Gadella *et al.*, 1994).

## Lipid signalling in human spermatozoa

### Lysophospholipid

**Figure 2.**
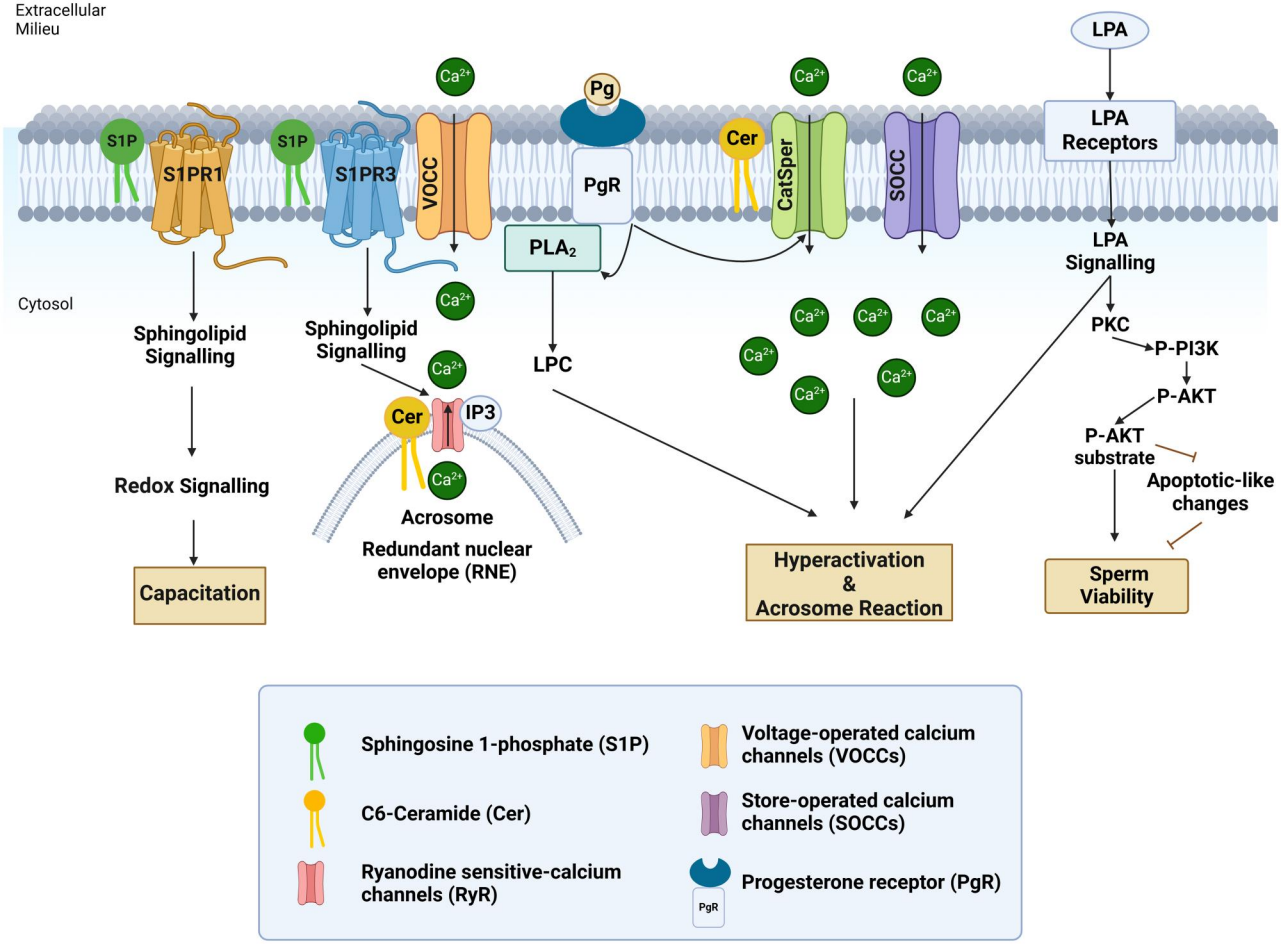
**Lipid-dependent signalling in spermatozoa.** This diagram illustrates the key lipid-mediated signalling pathways involved in sperm function, highlighting processes such as capacitation, acrosome reaction, hyperactivation motility regulation, and sperm viability. Progesterone (Pg) interaction with the progesterone receptor activates phospholipase A2, cleaving phosphatidylcholine to generate lysophosphatidylcholine, which triggers the acrosome reaction. Sphingosine 1-phosphate (S1P) binds to sphingosine 1-phosphate receptor 1 (S1PR1), activating redox signalling that induces capacitation-associated modifications. Additionally, S1P interacts with sphingosine 1-phosphate receptor 3 (S1PR3), activating Voltage-operated calcium channels, which initiate calcium entry. In addition, S1PR3-mediated signalling triggers intra-acrosomal calcium efflux from the Ryanodine sensitive-calcium channels (RyR) at the level of the acrosome and redundant nuclear envelope and calcium influx through store-operated calcium (SOCCs) and CatSper channels at the plasma membrane. The sustained calcium increase promotes hyperactivation and facilitates the acrosome reaction. Ceramide and Pg activate CatSper, RyR, and SOCCs to promote calcium mobilization towards the head and induce acrosome exocytosis. Lysophosphatidic acid (LPA) binds to LPA receptors (LPARs), activating the PI3K/AKT pathway to prevent apoptotic-like changes and maintain sperm viability. Moreover, LPA-LPAR signalling promotes the acrosome reaction. This diagram summarizes lipid-signalling molecules and their respective cascades, emphasizing their roles in modulating sperm behaviour and enhancing fertilization potential. This figure was created in BioRender. Lab, O. (2025) https://BioRender.com/a07t953.

As spermatozoa travel through the female reproductive tract, they must remain viable, completing capacitation and fertilizing the oocyte upon ovulation (Suarez and Pacey, 2006; Suarez, 2016). A significant process to sustain sperm viability is the activation of the phosphatidylinositol 3-kinase (PI3K)/protein kinase B pathway (Koppers *et al.*, 2011; Linn *et al.*, 2021). Disruption of this pathway leads to apoptotic-like changes, reducing sperm motility and viability, increasing lipid peroxidation, and causing DNA damage. Peroxiredoxins (PRDXs) scavenge ROS and regulate ROS-dependent signalling to protect spermatozoa against oxidative stress (Fernandez and O’Flaherty, 2018). PRDX6 possesses peroxidase and calcium-independent phospholipase A_2_ (iPLA_2_) and LPC acyltransferase activities (Chatterjee *et al.*, 2011; Li *et al.*, 2015; Fisher *et al*., 2016). PRDX6 iPLA_2_ activity maintains sperm viability by releasing LPA (Fernandez *et al.*, 2019). LPA is a bioactive lysophospholipid reported in somatic cells to be involved in cell proliferation and migration (Xiang *et al.*, 2020). LPA is synthesized from plasma membrane phospholipids by phospholipase D (PLD), converting them to PA, then to LPA by PLA_2_. Alternatively, PLA_2_ cleaves at the sn-2 position of the glycerol backbone of a phospholipid and thus produces an FA and one lysophospholipid, which is metabolized by PLD into LPA. LPA activates G protein-coupled receptors LPAR1-5 (Xiang *et al.*, 2020). Recent findings show that LPAR1, 3, 5, and 6 are present in the human sperm plasma membrane. Inhibition of LPAR1-3 impairs sperm viability by disrupting the PLC and PKC-regulated LPAR-PI3K-AKT survival pathway (Fig. 2) (Liao and O’Flaherty, 2023).

LPA, abundant in seminal plasma and follicular fluid, enhances human sperm motility and hyperactivation in a dose-dependent manner by boosting glycolysis and activating L-type calcium channels (Fig. 2) (Li *et al.*, 2022). Additionally, LPA can activate PKCα, which is implicated in the induction of AR (Garbi *et al.*, 2000) (Fig. 2), and promote actin polymerization (Delgado‐Buenrostro *et al.*, 2005) necessary for oocyte penetration. The specific LPARs involved in these processes are unclear, necessitating further research to clarify the molecular mechanisms of LPA-LPAR interactions and explore other functions of LPARs in human spermatozoa.

LPC is involved in AR by helping to destabilize the cell membrane to favour the exocytosis of the acrosomal content and facilitate the fusion process (Ehrenwald *et al.*, 1988). This destabilization can trigger the release of secretory PLA_2_ from human spermatozoa, producing LPC around the sperm (Lessig *et al.*, 2006). LPC, with lysophosphatidylethanolamine (LPE) and lysophosphatidylinositol (LPI), promotes the AR. In contrast, lysophospholipid inhibits AR induced by LPC, LPE, and LPI (Ehrenwald *et al.*, 1988). Building on these findings, previous research suggests that PLA_2_ may play a role in the interaction between sperm and the ZP of the egg (Takei *et al.*, 1984).

Riffo and Nieto (1999) showed that LPC enhanced sperm binding to the ZP, and was blocked by pre-treating sperm with pertussis toxin, inhibiting the AR (Riffo and Nieto, 1999). The authors found that LPC, but not PC, targets the ZP, as similar results occurred when applied to isolated ZP (Riffo and Nieto, 1999). Hence, LPC modulates sperm–ZP interactions by altering the ZP’s structure, facilitating sperm binding and triggering the AR.

### Lipid rafts

Lipid rafts are dynamic, cholesterol-, and sphingolipid-rich structures in the plasma membrane, crucial for cellular signal transduction and membrane organization (Simons and Toomre, 2000). Recent studies highlight their importance in fertilization, particularly in signalling, capacitation, and the AR (Table 2) (Cross, 2004; Gadella *et al.*, 2008; Nixon *et al.*, 2011).

**Table 2.** Changes in lipid raft organization and their embodied constituents in the events leading to fertilization.

Major findings	Role	Species	Source
Sterol loss during capacitation reduces lipid rafts, affecting CD59 and GM levels; flotillin-2 remains unchanged.	Sterol loss disrupts lipid raft organization and sperm function.	Human	Cross (2004)
GM1 localization shifts from the flagellum to the acrosomal region during capacitation; CD59 is also found in this region.	Lipid rafts play a role in sperm capacitation and AR.	Human	Nixon *et al.* (2011)
Controlled cholesterol efflux enhances capacitation; excessive cholesterol loss disrupts lipid diffusion and phosphorylation.	Cholesterol efflux is crucial for lipid raft organization and sperm capacitation	Boar	Shadan *et al.* (2004)
Capacitation leads to a change in concentration, not disintegration, of lipid rafts. The proteins are identified in DRMs.	Reorganization of lipid rafts during capacitation facilitates sperm-zona pellucida binding and AR	Human	Van Gestel *et al.* (2005)
Lipid raft clustering is crucial for plasma membrane-acrosomal membrane docking; SNARE complexes are essential for fusion.	Lipid raft clustering and SNARE complex formation are key for AR and zona pellucida binding.	Boar	Tsai *et al.* (2010)
Formation of new SNARE complexes during the acrosomal reaction, involving complexin 2.	SNARE complex rearrangement and lipid raft clustering drive the AR.	Boar	Tsai *et al.* (2012)

CD59, cluster of differentiation 59; GM1, ganglioside M1; AR, acrosome reaction; DRMs, detergent-resistant membranes; SNARE, soluble N-ethylmaleimide-sensitive factor attachment protein receptor.

Cross (2004) investigated how sterol loss during capacitation affects lipid rafts in human sperm (Cross, 2004) and found a 67% reduction in 3β-hydroxy sterols and significant decreases in CD59 and GM1 levels (34% and 31%, respectively) in lipid rafts, while flotillin-2 levels remained unchanged (Table 2). Preventing sterol loss preserved CD59 and GM1 in the raft fraction. Fluorescence microscopy showed that CD59 and GM1 are distributed across the sperm surface and are not associated with the same rafts. Flotillin-2 localized to the posterior head and midpiece. Additionally, sterol loss during capacitation disrupts lipid raft organization, impacting sperm function (Cross, 2004). GM1 labelling in non-capacitated spermatozoa was confined to the flagellum, while in capacitated sperm, it localized to the acrosomal region of the head (Nixon *et al.*, 2011) (Table 2). CD59 was found in the acrosomal region as well. Proteomic analysis identified lipid-raft-associated proteins with potential roles in sperm–oocyte interactions and cellular adhesion, including ACE, SPAM1, and ZPB1.

Shadan *et al.* (2004) examined the effects of cholesterol efflux on boar sperm capacitation, focusing on tyrosine phosphorylation (P-Tyr), lipid diffusion, and lipid raft organization. Their findings showed that controlled cholesterol efflux with MβCD enhanced capacitation, while excessive cholesterol loss disrupted lipid diffusion, destabilized lipid rafts, and suppressed phosphorylation (Shadan *et al.*, 2004) (Table 2). Notably, they also reported that during MβCD-mediated capacitation, GM1 gangliosides shifted from the sperm tail to clusters on the head, indicating dynamic reorganization (Shadan *et al.*, 2004). There is a critical window for cholesterol removal that promotes protein phosphorylation and the polarized migration of lipid rafts. Preferential loss from the non-raft pool likely triggers raft clustering at the sperm head’s anterior region (Shadan *et al.*, 2004).

During sperm capacitation, the aggregation of lipid microdomains plays a pivotal role in sperm-ZP binding. This clustering of lipid rafts facilitates the removal of cholesterol from non-raft regions. Van Gestel *et al.* (2005) demonstrated that capacitation shifted the membrane distribution of Caveolin-1 and Flotillin-1 (Table 2). Lipid analysis of detergent-resistant membranes (DRMs) from capacitated spermatozoa revealed that capacitation leads to the concentration of lipid rafts rather than their disintegration, as the lipid content (e.g. PC, SM, and cholesterol) in the DRM fraction remained unchanged (Table 2).

Additionally, proteomic analysis identified several key DRM proteins, categorized into two groups: (i) proteins likely involved in the primary binding to the ZP, such as fertilin beta, sp32 precursor, spermadhesin AQN-3 (Boerke *et al.*, 2008), and preproacrosin, and (ii) proteins involved in redox balance, including aldose reductase, superoxide dismutase (SOD), and peroxiredoxin 5 (Van Gestel *et al.*, 2005). These findings suggest that the reorganization of lipid rafts during capacitation may facilitate capacitation-specific signalling events and enhance sperm binding to the ZP. Moreover, membrane fusion proteins, including ADAM5 and LYPD4 (Wang *et al.*, 2020), SPAM-1 (Griffiths *et al.*, 2008), Sp17 and ACE (Nixon *et al.*, 2011), HSP60 and ZPB1 (Tanphaichitr *et al.*, 2015), and AK1 (Girouard *et al.*, 2008), are also found in lipid rafts. Furthermore, in boar spermatozoa, proteins such as ACR, ACRBP, and AWN were detected in lipid rafts after capacitation (Boerke *et al.*, 2008). These studies underscore the critical role of lipid rafts in ZP binding and the AR.

Lipid raft clustering plays a crucial role in the docking of the sperm plasma membrane to the OAM, a key step in sperm’s ability to penetrate the ZP of the oocyte. Tsai *et al.* (2010) demonstrated that raft-associated ternary trans-SNARE complexes, composed of VAMP-3 and syntaxin 1B from the plasma membrane and SNAP-23 from the acrosomal membrane, are essential for this fusion (Tsai *et al.*, 2010) (Table 2). Further studies using calcium ionophore-induced ARs revealed the formation of mixed vesicles that did not contain the previously identified trimeric SNARE complex (Tsai *et al.*, 2012) (Table 2). Instead, these MVs contained a novel SNARE complex involving syntaxin 3, SNAP-23, and VAMP-2, along with an additional SNARE-interacting protein, complexin 2 (Tsai *et al.*, 2012). These findings highlight the critical roles of lipid raft clustering and rearrangement, including the recruitment of SNARE proteins and complexin 2 into newly formed lipid-ordered microdomains, the assembly of a fusion-driving SNARE complex that mediates the Ca^2+^-dependent AR, the disassembly of the initial docking SNARE complex, and the recruitment of secondary zona-binding proteins to the sperm surface for ZP interaction (Tsai *et al.*, 2012).

### Sphingolipids in AR

Sphingolipids are integral to the fertilization processes in spermatozoa. Adding sphingomyelin to sperm suspensions slows cholesterol efflux, and exogenous sphingomyelinase and cell-permeable ceramide enhanced capacitation, AR, and sterol loss (Cross, 2000). These findings suggest that sphingomyelin slows sterol loss in spermatozoa while sphingomyelinase accelerates capacitation by promoting sterol loss and generating ceramide.

Suhaiman *et al.* reported that the bioactive sphingolipid S1P is crucial in triggering AR in human spermatozoa (Suhaiman *et al.*, 2010). This process involves a previously unidentified sphingolipid Gi-coupled receptor and increases cytosolic calcium levels by activating voltage-operated calcium channels (VOCCs) (Fig. 2). This receptor was recently reported by our group as S1PR3 (Serafini & O’Flaherty, 2025). The increase in cytosolic calcium would activate PLC, leading to the production of IP_3_ and activating ryanodine sensitive-calcium channels (RyR) located in the acrosome and the redundant nuclear envelope of the spermatozoon (Vaquer *et al.*, 2020) (Fig. 2). Emptying of calcium stores activates store-operated calcium channels (SOCCs) at the plasma membrane, contributing to calcium mobilization towards the sperm head and acrosome exocytosis (Fig. 2) (Darszon *et al.*, 2001; Breitbart, 2003). Moreover, Suhaiman *et al.* reported that sphingosine kinase 1 (SphK1) redistributes to the acrosomal region and is activated upon phorbol ester treatment, a known PKC activator (Suhaiman *et al.*, 2010). These findings provide the first evidence of S1P production in human sperm during AR (Fig. 2), highlighting its role and suggesting new lipid-signalling pathways in this process.

Vaquer *et al.* (2020) identified several enzymes involved in sphingolipid metabolism in spermatozoa, including neutral sphingomyelinase and ceramide synthase (Vaquer *et al.*, 2020). Their findings revealed that both externally added and endogenous ceramide act in concert with progesterone to increase intracellular calcium levels through RyR, the CatSper calcium channel, and SOCCs, ultimately promoting exocytosis (Fig. 2) (Vaquer *et al.*, 2020).

### Sphingolipid in sperm capacitation

Despite the importance of sphingolipids in the AR, many aspects of sphingolipid signalling remain unclear, particularly during capacitation. Recently, we investigated sphingolipid signalling in human sperm capacitation (Serafini and O’Flaherty, 2025). We found that cell-permeable sphingosine and ceramide alone promoted capacitation-related changes, including enhanced P-Tyr, hyperactive motility, and progesterone-induced AR (Serafini and O’Flaherty, 2025). We demonstrated for the first time that the ABCC1 transporter exports intracellularly produced S1P, generated by SphK1, to engage with Gi-coupled S1PRs. We identified S1PR1, not S1PR3, as the receptor involved in P-Tyr during capacitation (Serafini and O’Flaherty, 2025) (Fig. 2). As mentioned, S1PR3 is involved in the AR (Suhaiman *et al.*, 2010; Serafini and O’Flaherty, 2025). Hence, S1P receptors may be differentially available to interact with S1P during capacitation AR.

We recently showed that S1P binding to S1PR1 activates the PI3K-AKT pathway, triggering oxidative stress processes, including NO production (see Graphical Abstract). This pathway is crucial for driving capacitation-related modifications. Before our study, the regulation of NO production through this pathway was unknown (Breitbart, 2003). Additionally, sphingosine and ceramide promote the production of O_2_^•−^. The extracellular addition of SOD prevented sphingosine- and ceramide-dependent capacitation, indicating they stimulate the production of O_2_^•−^ from the alleged sperm oxidase located on the extracellular leaflet of the plasma membrane. This O_2_^•−^ generated also activates a kinase novel to the field of andrology, protein kinase R (PKR). PKR, ceramide kinase, and PKC are responsible for translocating and activating SphK1, which is necessary to promote S1P production for sperm capacitation.

Sphingosine, ceramide, S1P, and C1P are essential for human sperm capacitation. Our recent research (Serafini and O’Flaherty, 2025) reveals a new mechanism through which these sphingolipids support capacitation, offering insights into their role in sperm function and potential lipid profile imbalances in infertile males that could impact fertilization potential.

## Conclusion

The fusion of sperm and egg is a critical event in sexual reproduction. Lipids are integral to maintaining sperm integrity, controlling membrane fluidity, and forming functional membrane domains, all essential for sperm function and successful fertilization. These lipid components are involved at various stages of sperm development, and their profile continuously evolves, from spermatogenesis and sperm maturation to the binding and fusion with the egg. Rather than acting in isolation, lipid classes interact in complex, coordinated pathways through processes of synthesis, metabolism, and interconversion. This review highlights the role of different lipid types in sperm viability and key fertilization events, particularly capacitation and the AR.

Despite the growing body of research, significant gaps remain in our understanding of lipid signalling in spermatozoa, particularly regarding the lipid profiles in both fertile and infertile male patients, and the subsequent positive or negative impacts it presents, warranting further investigation. Clinically, advancing our knowledge of lipid dynamics in sperm function could lead to the development of novel diagnostic tools and therapeutic approaches for male infertility. Additionally, innovative techniques and methods may shed light on lipid-regulated fertilization processes and the potential consequences of disrupted lipid levels on male fertility in mammals.

## Data Availability

No new data were generated or analysed in support of this research.
